# Gut and skin microbial dysbiosis correlate with systemic inflammation and pruritus in immunological non-responders

**DOI:** 10.3389/fmicb.2026.1758111

**Published:** 2026-04-01

**Authors:** Jiahe Li, Liran Xu, Xue Ding, Nao Qiu, Jingyu Yue

**Affiliations:** 1 Department of the First Clinical Medical College, Henan University of Chinese Medicine, Zhengzhou, China; 2 Department of AIDS Clinical Research Center, The First Affiliated Hospital of Henan University of Chinese Medicine, Zhengzhou, China; 3 Department of Medical, The First Affiliated Hospital of Henan University of Chinese Medicine, Zhengzhou, China

**Keywords:** gut microbiome, skin microbiome, immunological non-responders, microbial dysbiosis, people living with HIV, pruritus, systemic inflammation

## Abstract

**Introduction:**

Among people living with human immunodeficiency virus (PLWH), those who exhibit immunological non-responders (INR) are highly susceptible to developing pruritus. The aim of this study was to examine the relationships among pruritus, alteration of the gut and skin microbiomes, and systemic inflammation in PLWH with INR.

**Methods:**

Thirty-three PLWH with INR were enrolled and divided into Pruritus (*n* = 18) and Control (*n* = 15) groups. All participants met the defining criterion of a low CD4+ T cell count ( ≤ 350 cells/μl). We performed 16S rRNA gene sequencing of fecal and skin samples, and measured plasma IL-1β and IL-10 levels.

**Results:**

Microbiome analysis revealed specific, bidirectional patterns of microbial dysbiosis. Specifically, the skin microbiome of the Pruritus Group exhibited significantly greater microbial richness (Chao1 and Faith's Phylogenetic Diversity indices, *P* < 0.01), coupled with significantly lower representation of the potentially protective genus *Bacillus* (*adjusted P*<*0.05*), compared with that of the INR Control Group. Conversely, the gut microbiome of the Pruritus Group exhibited significantly lower alpha diversity *(adjusted P*<*0.05)*. Furthermore, we identified a significant positive correlation between levels of plasma pro-inflammatory cytokine IL-1β and the relative abundance of the opportunistic gut genus *Veillonella* (*adjusted P*<*0.05*).

**Conclusions:**

Pruritic PLWH-INRs exhibit skin microbial hyper-richness, Bacillus depletion, and reduced gut diversity, suggesting a systemic inflammatory basis linked to gut-skin dysbiosis. These findings provide new insights into the pathological process, offering a potential foundation for future microbiome-targeted therapeutic strategies as novel management avenues.

## 1 Introduction

Human immunodeficiency virus (HIV) infection can lead to chronic immune activation and inflammation, even with effective antiretroviral therapy (ART) (Deeks et al., 2013). ART is crucial for viral suppression and immune reconstruction; however, some patients experience incomplete immune recovery, a condition known as immunological non-responders (INR). This persistent immune dysfunction can contribute to various complications, including dermatological issues (Chimbetete et al., 2023). Pruritus is a highly prevalent and often debilitating symptom experienced by people living with HIV (PLWH). Its chronic nature profoundly impairs quality of life, frequently resulting in secondary clinical sequelae, including excoriation, skin damage, chronic sleep disturbance, and psychological distress (Kaushik et al., 2014). Skin microbial dysbiosis, or an imbalance in these microbial communities, has been linked to various inflammatory and autoimmune diseases. Specifically, skin microbial dysbiosis refers to a state where the composition, diversity, or functional balance of the skin microbiota is disrupted (Chen et al., 2026). This often involves the depletion of beneficial commensal bacteria and an increase in opportunistic microorganisms, leading to impaired skin barrier integrity and heightened local immune responses. The etiology of pruritus in PLWH is complex and multifactorial, encompassing a broad spectrum of causes that include opportunistic infections, adverse drug reactions, and specific inflammatory dermatoses. Critically, for a significant proportion of PLWH, the underlying pathomechanisms remain elusive, which impedes effective treatment. Recent evidence has highlighted the critical role of the microbiome in maintaining immune homeostasis and overall health (de Vos et al., 2022). The human body is a complex ecosystem inhabited by trillions of microorganisms, including bacteria, fungi, and viruses, which reside on the skin, in the gut, and in other bodily sites. The gut microbiome is a key player in modulating systemic immunity, as it influences the maturation and function of immune cells (Wiertsema et al., 2021). The skin microbiome is essential for skin barrier integrity and local immune responses (Zhang et al., 2024). Dysbiosis, or an imbalance in these microbial communities, has been linked to various inflammatory and autoimmune diseases (Wang et al., 2024).

In the context of HIV infection, it is increasingly recognized that both the gut and skin microbiomes undergo significant changes (Ogai et al., 2023). Immunosuppression and chronic inflammation can disrupt the delicate balance of these microbial ecosystems. Although previous studies have shown that the skin microbiome's composition is altered in PLWH, they have not defined the specific relationship of the skin microbiome with pruritus, especially in those with INR. Furthermore, the interplay between the gut and skin microbiomes, and its contribution to chronic inflammatory skin conditions such as pruritus, in PLWH warrants investigation.

In this study, we aimed to comprehensively analyze the gut and skin microbiomes of PLWH with INR who present with pruritus, thereby elucidating the microbial and systemic inflammatory factors associated with this debilitating symptom. By comparing microbial profiles and inflammatory cytokine levels between PLWH who exhibit INR with and without pruritus, we sought to determine if there is a distinct microbial dysbiosis associated with pruritus. Our findings provide new insights into the pathological process of pruritus in this vulnerable patient population, offering a potential foundation for future research into microbiome-targeted therapeutic strategies.

## 2 Materials and methods

### 2.1 Ethical considerations

This study received ethical approval from the Research Ethics Committee of the First Affiliated Hospital of Henan University of Chinese Medicine (Approval No. 2025HL-497). All procedures adhered to the principles outlined in the Declaration of Helsinki. Prior to participation, all individuals were provided with written information about the study, and written informed consent was obtained from each participant.

### 2.2 Study population

This study was part of a cross-sectional study conducted at the First Affiliated Hospital of Henan University of Chinese Medicine, a designated hospital for ART for HIV/AIDS in Henan Province (China).

To exclude the baseline impact of HIV infection and long-term immunodeficiency on the microbiome and to focus specifically on the mechanisms underlying pruritus, we selected a INR Control Group of INRs without pruritus. These controls were matched for key clinical characteristics, including CD4+ T cell counts and viral load. According to the 2023 edition of the “Consensus on diagnosis and management of immunological non-responders in HIV infection,” the diagnostic criteria for INR include receipt of ART for >4 years, a peripheral blood viral load below the detection limit (viral load < 50 copies/ml) for more than 3 years, and a CD4+ T cell count persistently < 350/μl. Other possible causes of long-term low CD4+ T cell counts should be excluded, such as known types of immune deficiency or immune deficiency, chronic viral infections, hematological neoplastic diseases, and long-term use of immunosuppressive drugs.

The inclusion criteria for the study included (1) an HIV infection diagnosis, (2) a score of ≥4 on the visual analog scale or not, and (3) pruritus without systemic treatment (including antibiotics, probiotics, antifungals, or corticosteroids) in the past month. The exclusion criteria included (1) non-compliance with medical advice; (2) pregnant or lactating women; (3) severe organic diseases such as cirrhosis, renal failure, heart failure, or pneumonia; and (4) individuals who had undergone surgery within the past 3 months.

From January to June 2025, 33 PLWH with INR were enrolled and divided into the Pruritus Group and the INR Control Group based on the presence of pruritus. Prior to the start of the study, all enrolled patients formally signed an informed consent form.

The recruitment process presented significant challenges due to the stringent inclusion criteria for INRs, particularly those complicated by chronic pruritus. Specifically, the criteria required a history of long-term antiretroviral therapy (ART >4 years) with sustained viral suppression yet incomplete immune reconstitution. Consequently, this study was designed as a preliminary exploratory analysis, aimed at generating theoretical hypotheses to inform future large-scale cohort studies.

### 2.3 Sample collection

#### 2.3.1 Skin samples

Skin swabs were collected as described previously (Ogai et al., 2022). We collected skin microbiota samples from four different body sites: right anterior forearm, lateral lower limbs, abdomen, back. This approach allowed us to obtain a more comprehensive profile of the skin microbiome and reduced the variability caused by site-specific differences. At each site, a 5^*^5 cm^2^ area was swabbed six to eight times using a swab presoaked in sterile normal saline to ensure adequate collection of microbial DNA. Samples from these four sites were pooled into a single tube for each participant to represent the overall skin microbiota status. Each swab head was cut and placed in a cryogenic vial containing cryopreservation medium before storage at −80 °C until DNA extraction. Plasma samples were collected in the morning with ethylenediaminetetraacetic acid-coated anticoagulant tubes. Samples were then centrifuged at 1,000 × *g* for 15 min, and supernatants were aliquoted into cryovials for storage at −80 °C.

#### 2.3.2 Fecal samples

Patients were required to provide fecal samples. Trained laboratory physicians used disposable sterile cotton swabs to gently collect ≥1 g of feces by swiping over the surface. Each swab was then placed in a sterile storage tube and immediately frozen at −80 °C.

### 2.4 Cytokine analysis

Cytokine levels were measured using enzyme-linked immunosorbent assay (Proteintech, IL-10 and IL-1β, Rosemont, IL, USA) according to the manufacturer's instructions.

### 2.5 DNA extraction

Total genomic DNA from skin and gut microbiome samples was extracted using cetyltrimethylammonium bromide, following the manufacturer's instructions, and stored at −20 °C until further analysis. The quantity and quality of extracted DNA were measured using a NanoDrop NC2000 spectrophotometer (Thermo Fisher Scientific, Waltham, MA, USA) and agarose gel electrophoresis, respectively.

### 2.6 16S rRNA gene amplicon sequencing

Polymerase chain reaction amplification of the bacterial 16S rRNA gene V3–V4 region was performed using primers 338F (5′-ACTCCTACGGGAGGCAGCA-3′) and 806R (5′- GGACTACHVGGGTWTCTAAT-3′). Amplicons were purified with Vazyme VAHTS DNA Clean Beads (Vazyme, Nanjing, China) and quantified using a Quant-iT PicoGreen dsDNA Assay Kit (Invitrogen, Carlsbad, CA, USA). After individual quantification, amplicons were pooled in equal amounts, and paired-end 2,250 bp sequencing was performed using the Illumina NovaSeq platform with a NovaSeq 6000 SP Reagent Kit at Shanghai Personal Biotechnology (Shanghai, China).

### 2.7 Sequencing analysis

Microbiome bioinformatics analysis was performed with QIIME2 2024.5 (QIIME2 Development Team) according to the official tutorials with slight modifications (Bolyen et al., 2019). Briefly, raw sequence data were demultiplexed using the demux plugin, then primers were excluded with the cutadapt plugin (Martin, 2011) Sequences were then quality-filtered, denoised, and merged, and chimeras were removed using the DADA2 plugin (Callahan et al., 2016).

### 2.8 Bioinformatics and statistical analysis

Sequencing data analysis was performed using QIIME2 and R v4.3.3 (R Foundation for Statistical Computing, Vienna, Austria). Amplicon sequence variant (ASV)-level alpha and beta diversity indices were calculated using the ASV table in QIIME2. Significant differences in microbiota structure among groups were assessed using QIIME2. Venn diagrams were generated to visualize the shared and unique ASVs among samples or groups using R package “VennDiagram,” based on the occurrence of ASVs across samples and groups regardless of their relative abundance (Zaura et al., 2009) Random forest analysis (RFA) was used to discriminate samples from different groups in QIIME2.

### 2.9 Correlation analysis between the microbiota and cytokines

Correlation *R*-value and *P*-value matrices were calculated in R to analyze the correlation between the two microbiota groups and cytokine expression using CorHeatmap. We analyzed the correlation between the top 20 genera and expression of the cytokines interleukin (IL) 10 and IL-1β in the HIV infection (Control) and HIV infection with pruritus (Pruritus) groups.

### 2.10 Statistical analysis

Statistical analyzes were performed using R (v4.3.3). The differential abundances of bacterial taxa at different levels (i.e., phylum, class, order, family, and genus) in the Control, Pruritus, and healthy (Normal) groups were calculated as averages. Differences in alpha diversity between the three groups were compared using the Kruskal–Wallis test, while differences in beta diversity were assessed using Bray–Curtis distances. To understand the differences in the metabolomic profiles of the Control and Pruritus Groups, we performed multivariate statistical analyzes for ASVs and operational taxonomic units, and employed Venn plots and RFA. RFA was employed as a supervised machine learning classification algorithm to identify microbial taxa that most effectively discriminate between the Pruritus and INR Control Groups. By constructing a multitude of decision trees, the model ranked bacterial genera based on their “importance scores” (Mean Decrease Gini), thereby facilitating the identification of potential microbial biomarkers associated with the pruritic state in PLWH with INR. Correlation analyzes for the cytokines and microbiota were conducted using Spearman's correlation coefficient. *P*-values < 0.05 were considered statistically significant. To control for the inflation of Type I error due to multiple comparisons in microbiome and correlation analyzes, *P*-values were adjusted using the Benjamini–Hochberg False Discovery Rate (FDR) method. Adjusted *P*-values (also referred to as *q*-values) < 0.05 were considered statistically significant.

## 3 Results

### 3.1 Baseline characteristics

The Pruritus Group (*n* = 18) had a mean CD4+ T cell count of 282.83 ± 42.71 cells/μl, while the INR Control Group (*n* = 15) had a mean of 278.73 ± 62.91 cells/μl. The nearly identical average CD4+ T cell counts for both groups fell within the typical INR range (350 cells/μl), confirming that all participants were immunocompromised. The mean age was similar between the two groups, at 54.94 years in the Pruritus Group and 53.27 years in the INR Control Group. The biological sex distribution was also comparable, with a male majority in both groups: 61.1% in the Pruritus Group and 53.3% in the INR Control Group.

### 3.2 Skin microbiota composition

**Figure 1:**
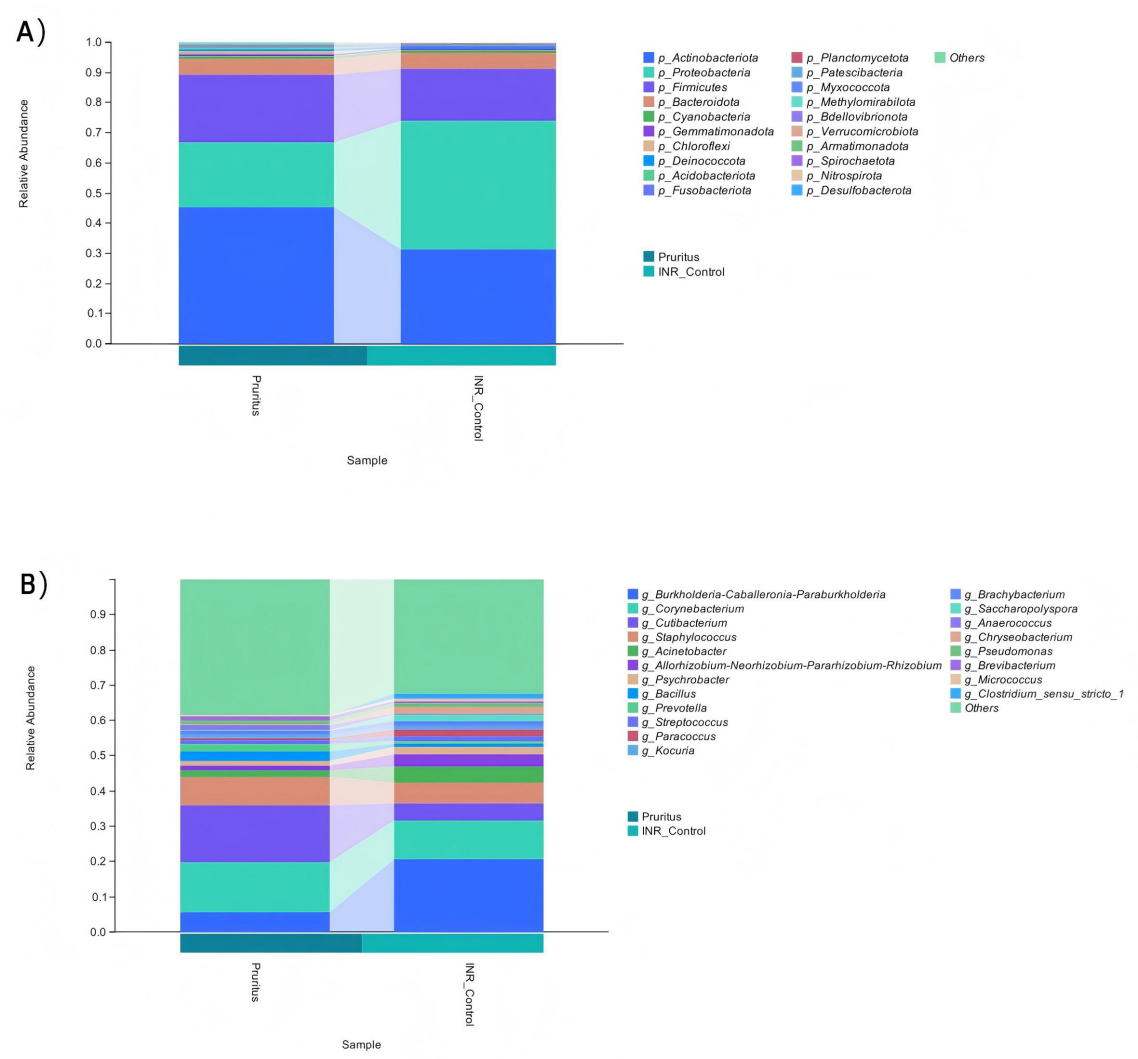
Relative abundance of microbiome communities in PLWH. **(A)** Skin microbiome at the Phylum level. **(B)** Skin microbiome at the Genus level. “Others” includes taxa with a relative abundance < 1%.

At the phylum level, the skin microbiome of both patient groups was primarily composed of three major phyla: *p_Actinobacteriota, p_Proteobacteria, and p_Firmicutes* (Figure 1A). In the INR Control Group, *p_Actinobacteriota* and *p_Firmicutes* comprised significantly lower proportions than in the Pruritus Group. Conversely, *p_Proteobacteria* were relatively less abundant in the Pruritus Group than in the INR Control Group.

At the genus level, the category “Others” accounted for the largest proportion in both groups (Figure 1B). *g_Burkholderia-Caballeronia-Paraburkholderia, g_Corynebacterium, g_Cutibacterium*, and *g_Staphylococcus* were abundant skin commensal genera in both groups. *g_Burkholderia-Caballeronia-Paraburkholderia* and *g_Corynebacterium* were less abundant in the Pruritus Group than in the INR Control Group. Conversely, *g_Cutibacterium* and *g_Staphylococcus* were less abundant in the INR Control Group than the Pruritus Group.

**Figure 2:**
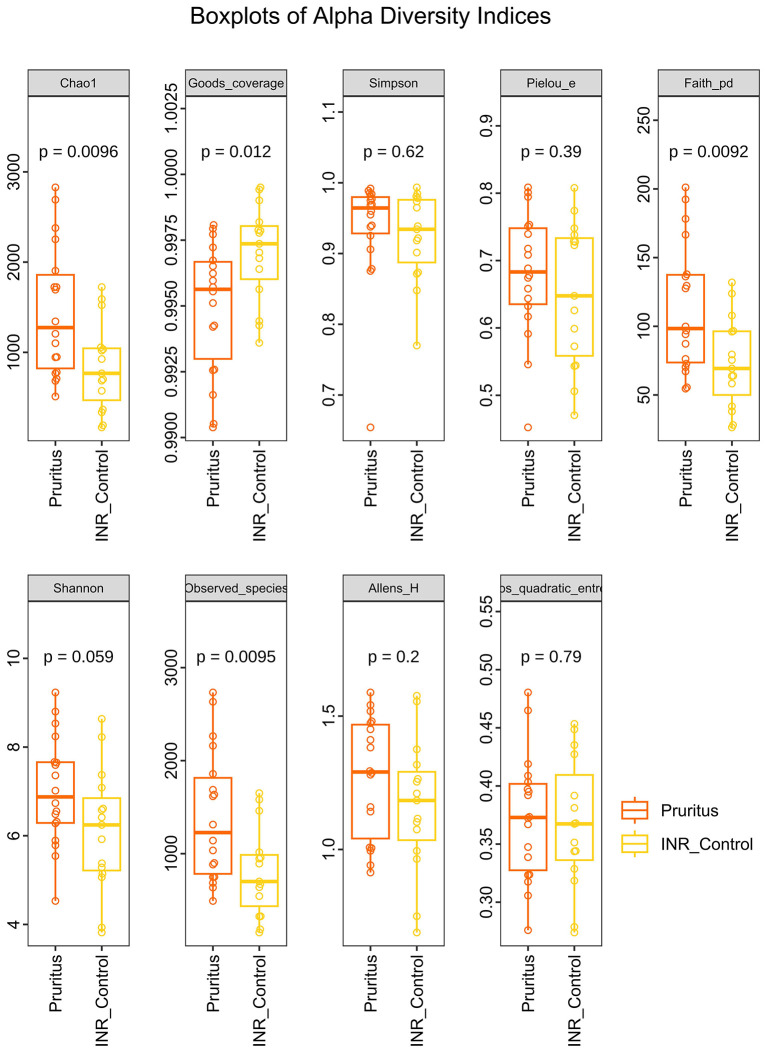
Alpha Diversity indices of the skin microbiota in PLWH with and without pruritus. *P*-values were calculated using the Kruskal–Wallis test. Horizontal lines in boxplots represent the median, while boxes indicate the interquartile range (IQR).

When assessing the diversity of the skin microbiome (Figure 2), we found the richness indices Chao1, *Observed_species*, and *Faith_pd* were significantly higher in the Pruritus Group (*P* < 0.01), whereas the Shannon and Simpson evenness indices did not statistically differ between groups.

**Figure 3:**
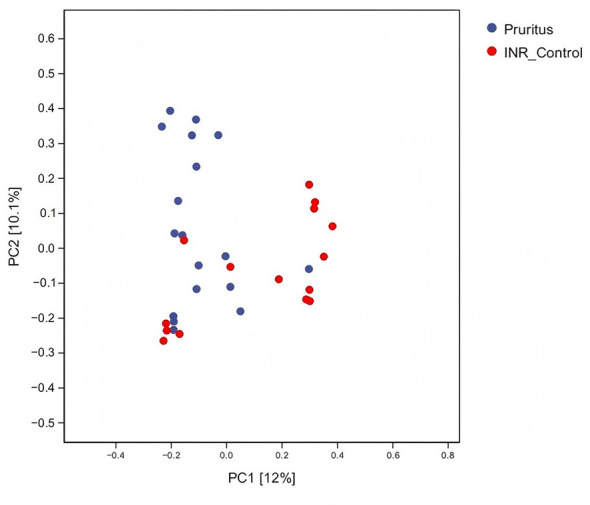
Principal Coordinate Analysis (PCoA) of skin microbiota beta diversity based on Bray-Curtis distances. Each point represents an individual sample (Blue: Pruritus group, *n* = 18; Red: INR Control group, *n* = 15).

Beta diversity analysis using Bray–Curtis distance (Figure 3) showed that the sample points in the Pruritus Group were more widely distributed along the PC1 axis, with a slight tendency toward the negative PC1 and negative PC2 axes. This suggests greater heterogeneity in the skin microbial community structure in the Pruritus Group, indicating greater inter-individual variability. The INR Control Group samples tended to cluster toward the center and upper right of the plot, with relatively shorter distances between them. The sample points from the two groups did not form completely distinct clusters.

**Figure 4:**
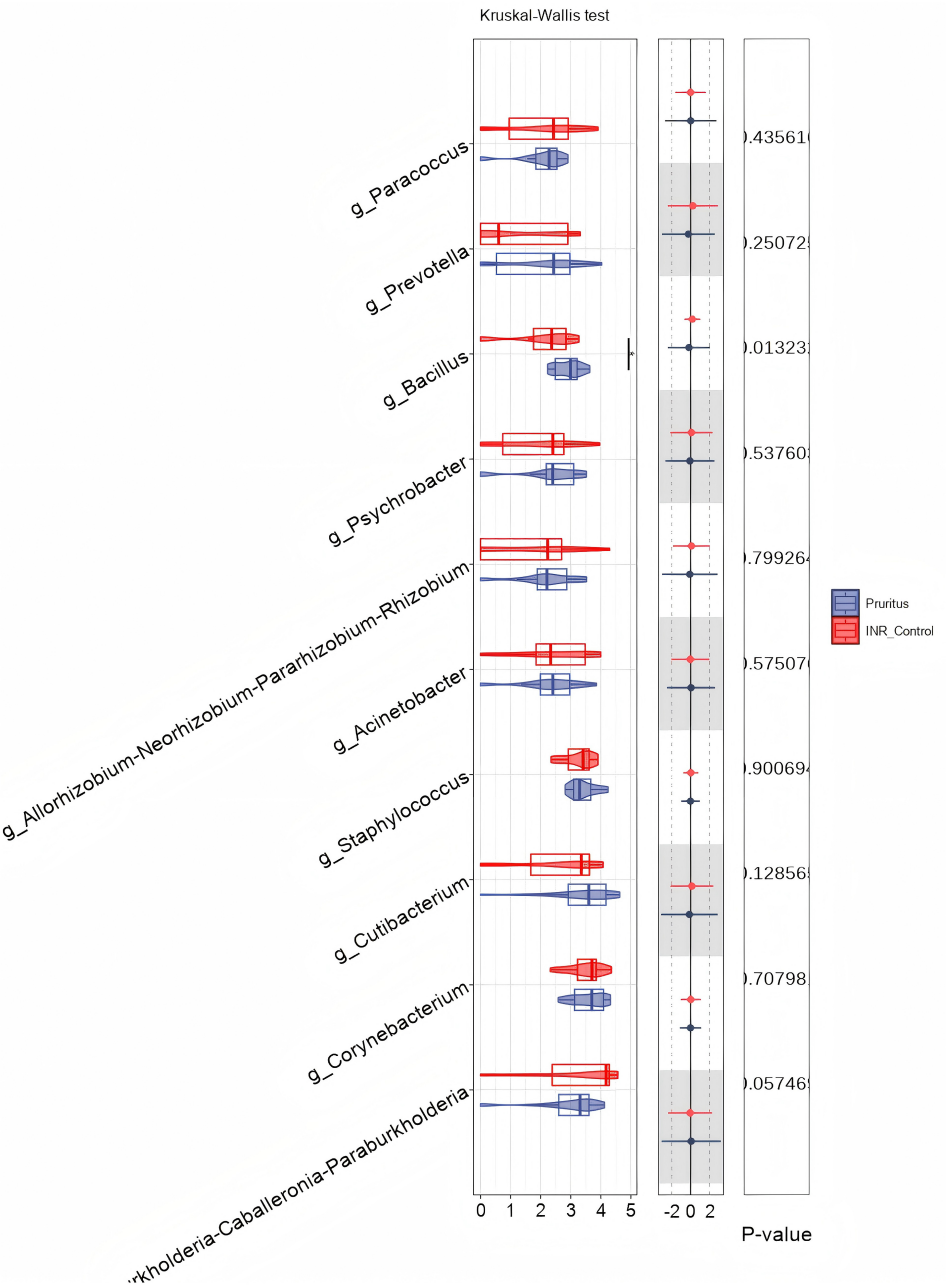
Relative abundance of skin microbiota at the Genus level shown via violin plots. *P*-values are based on the Kruskal–Wallis test. The width of the violin represents the density of data points at different abundance levels.

To determine species differences between the skin microbiomes of the two groups, we performed a Kruskal–Wallis test at the genus level. Among the listed genera, *g_Bacillus* was more abundant in the INR Control Group (red) than in the Pruritus Group (blue; adjusted *P* < 0.05; Figure 4). Genera such as *g_Cutibacterium, g_Staphylococcus, g_Corynebacterium*, and *g_Prevotella* tended to differ in abundance between the groups but the differences did not reach statistical significance (*P* ≥ 0.05).

**Figure 5:**
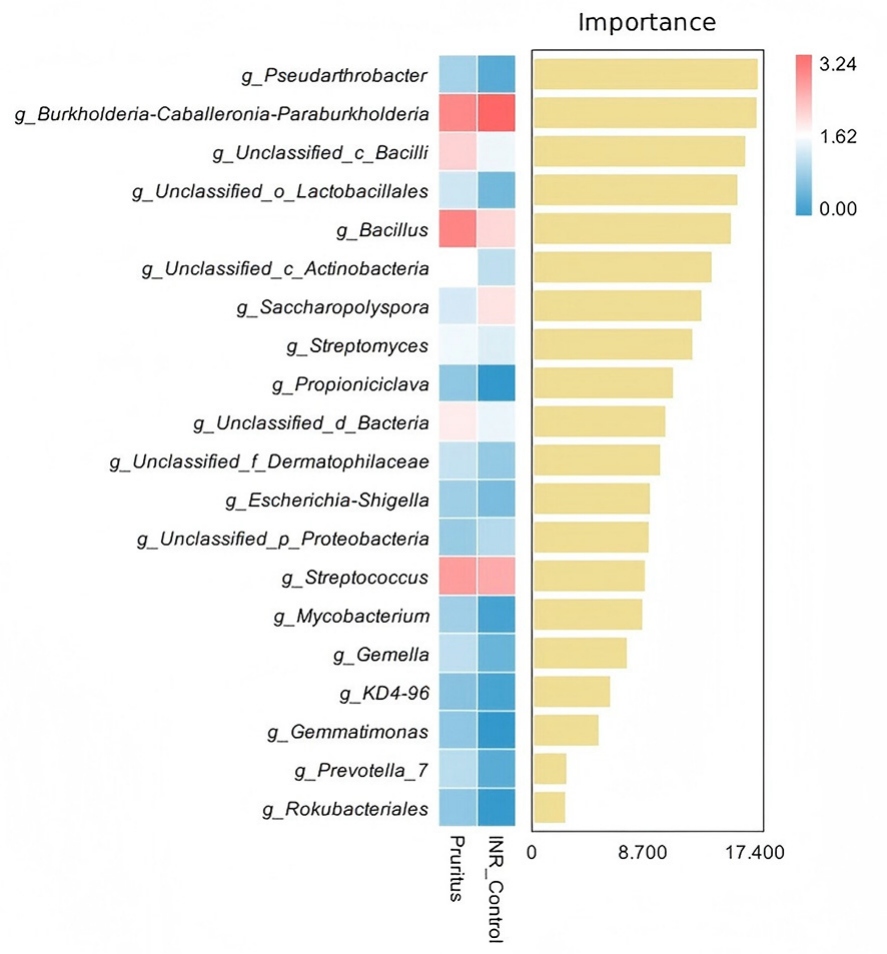
Random Forest Analysis (RFA) identifying discriminative skin microbial genera. Taxa are ranked by their Importance Score (Mean Decrease Gini). The heatmap indicates relative enrichment (Red: High; Blue: Low) in the Pruritus (*n* = 18) vs. INR Control (*n* = 15) groups.

The RFA indicated that the top five skin microbial genera ranked by importance scores were *g_Pseudarthrobacter, g_Burkholderia-Caballeronia-Paraburkholderia, g_Unclassified_c_Bacilli, g_Unclassified_o_Lactobacillales*, and *g_Bacillus* (Figure 5). Regarding abundance trends, the highest-ranked genera (including *g_Pseudarthrobacter, g_Burkholderia-Caballeronia-Paraburkholderia, g_Unclassified_c_Bacilli, and g_Bacillus*) were predominantly enriched in the Pruritus Group. In contrast, the genera ranked 6th and 7th (*g_Unclassified_c_Actinobacteria* and *g_Saccharopolyspora*) showed significant enrichment in the INR Control Group. Overall, the abundance differences among the top-ranked genera demonstrated a pronounced polarized trend between the two groups. Additionally, other notable genera identified by the model included *g_Cutibacterium* and *g_Rothia*. Specifically, *g_Cutibacterium* (ranked 14th) exhibited marked enrichment in the Pruritus Group, a pattern consistent with several top-ranked biomarkers such as *g_Bacillus*. This positions g_Cutibacterium as a significant microbial marker for identifying the pruritic state in PLWH with INR. Conversely, *g_Corynebacterium* was distinctly enriched in the INR Control Group, highlighting its potential role as a characteristic taxon of the non-pruritic skin microenvironment in this population.

### 3.3 Gut microbiota composition

**Figure 6:**
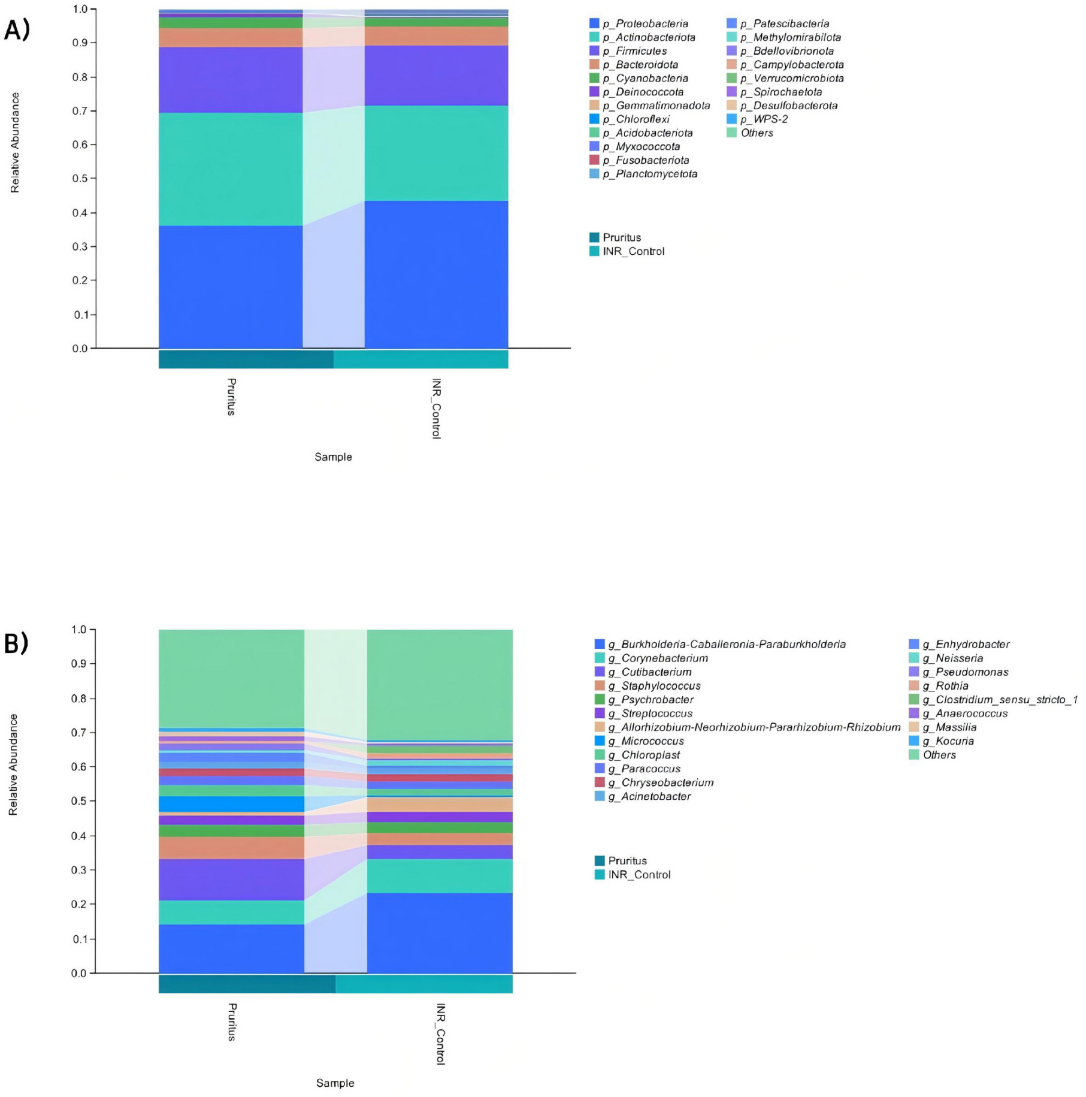
Relative abundance of gut microbiota communities in PLWH at the **(A)** Phylum and **(B)** Genus levels. Proportions are calculated as averages for each group.

At the phylum level, the gut microbiota of both groups were primarily composed of *p_Proteobacteria, p_Actinobacteriota, p_Firmicutes, and p_Bacteroidota* (Figure 6A). *p_Proteobacteria* was significantly less abundant in the Pruritus Group than the INR Control Group. Conversely, *p_Actinobacteriota* and *p_Firmicutes* were less abundant in the INR Control Group than the Pruritus Group.

At the genus level, “Others” accounted for the largest proportion in the gut samples of both groups. The proportions of *g_Burkholderia-Caballeronia-Paraburkholderia* and *g_Corynebacterium* were relatively less abundant in the Pruritus Group than in the INR Control Group, whereas *g_Cutibacterium* and *g_Staphylococcus* were less abundant in the INR Control Group than the Pruritus Group.

**Figure 7:**
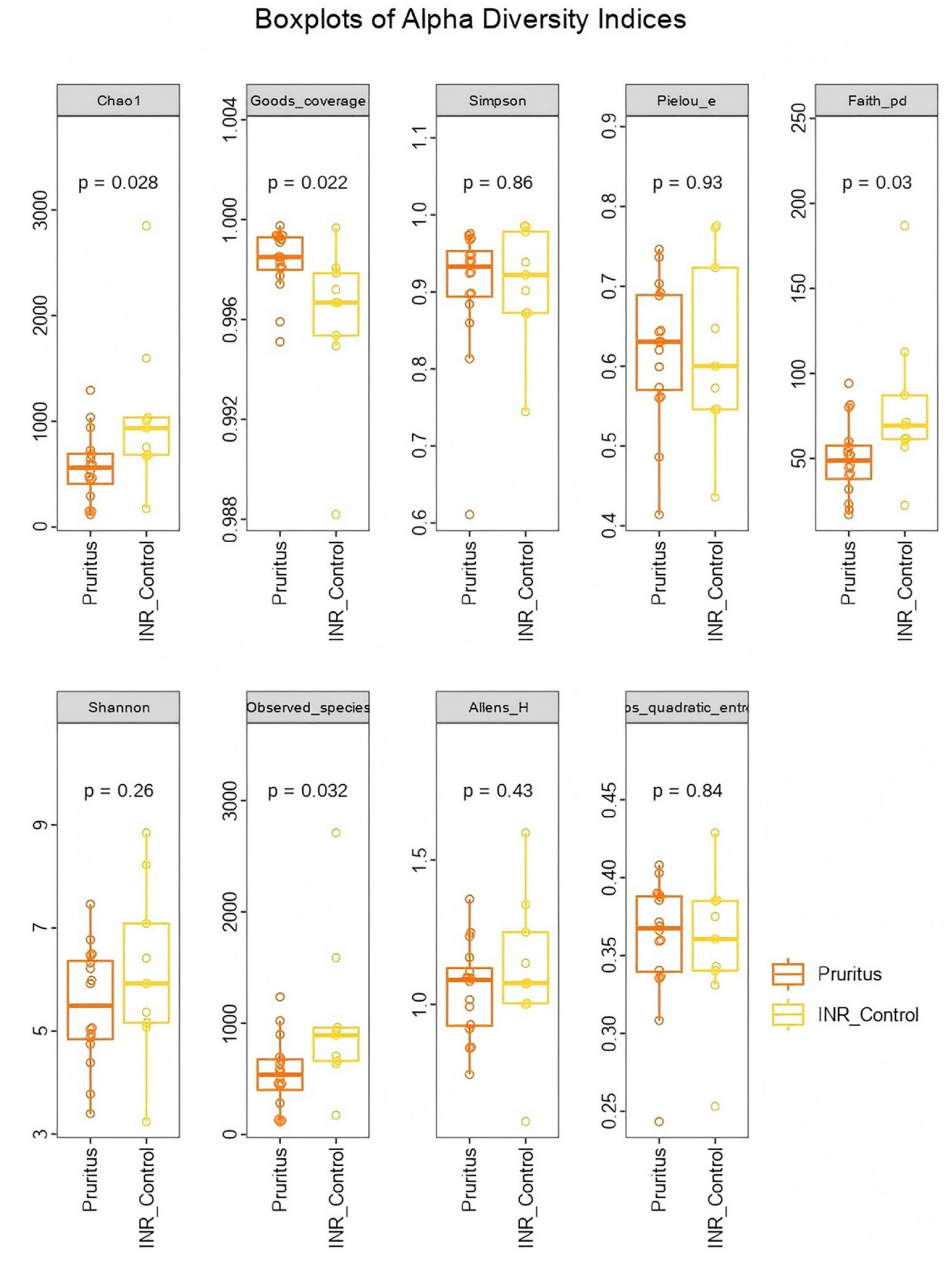
Alpha Diversity indices of the gut microbiota. Indices include species richness, phylogenetic diversity, and Observed_species. *P*-values were determined by the Kruskal–Wallis test.

Alpha diversity analysis revealed that the species richness, phylogenetic diversity, and number of species in the gut in the Pruritus Group were significantly lower than those in the INR Control Group (*adjusted P*<*0.05*; Figure 7). Although the coverage was high in both groups (99.6%), the Pruritus Group showed significantly lower coverage. No significant differences were observed in any other indices.

**Figure 8:**
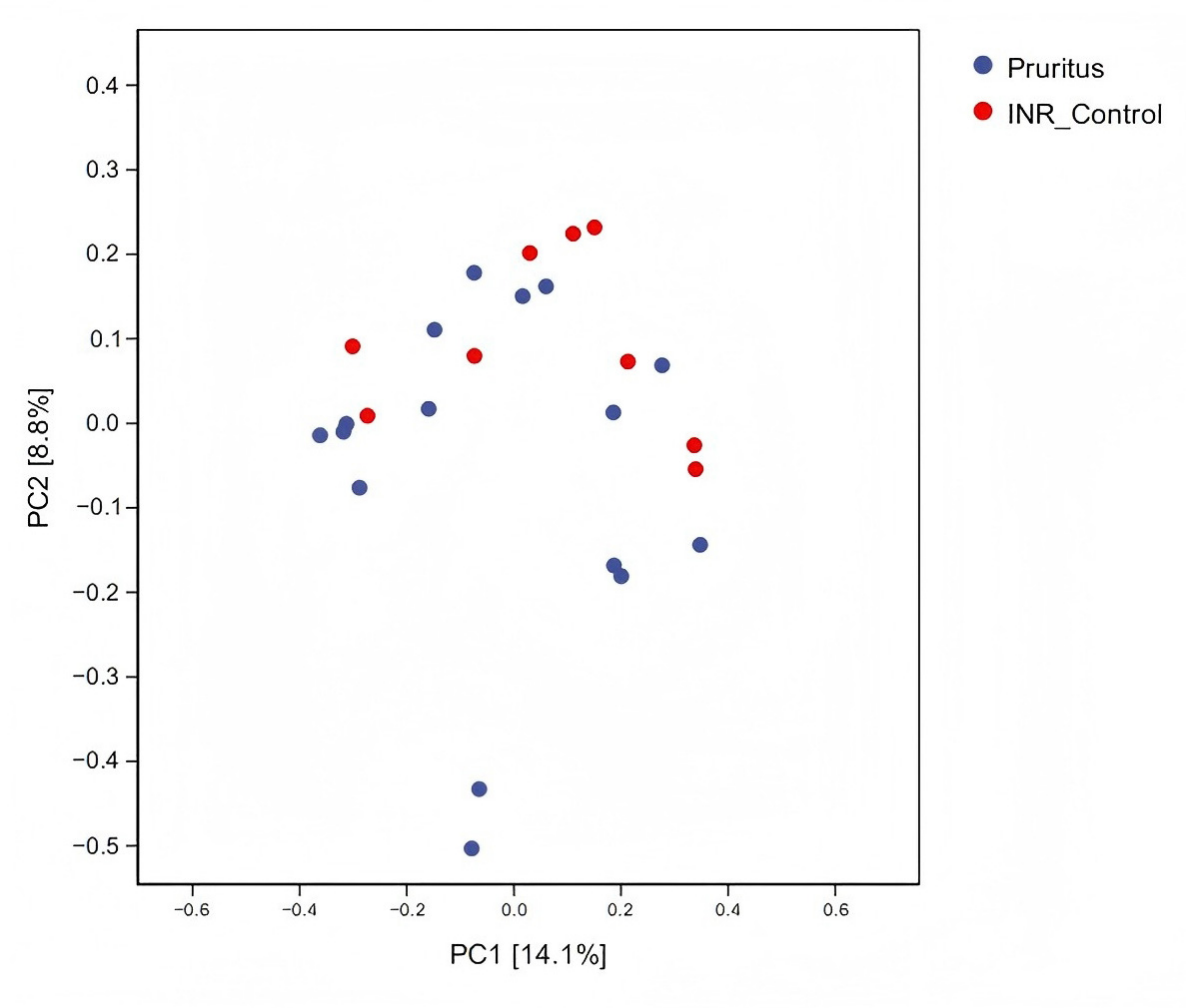
PCoA Plot of Beta Diversity of the gut microbiota communities in PLWH. The analysis is based on Bray-Curtis distances, reflecting the heterogeneity of the gut microbial structure.

Beta diversity analysis using Bray–Curtis distance showed that the sample points of the Pruritus Group appeared more dispersed than those of the INR Control Group, indicative of greater variance (Figure 8). Thus, the gut microbial community structure among patients in the Pruritus Group had higher heterogeneity, suggesting greater inter-individual variability. The community structure of the gut microbiome in the INR Control Group was relatively tighter and more stable, with smaller individual differences. The sample points from the two groups were not completely distinct on the plot.

**Figure 9:**
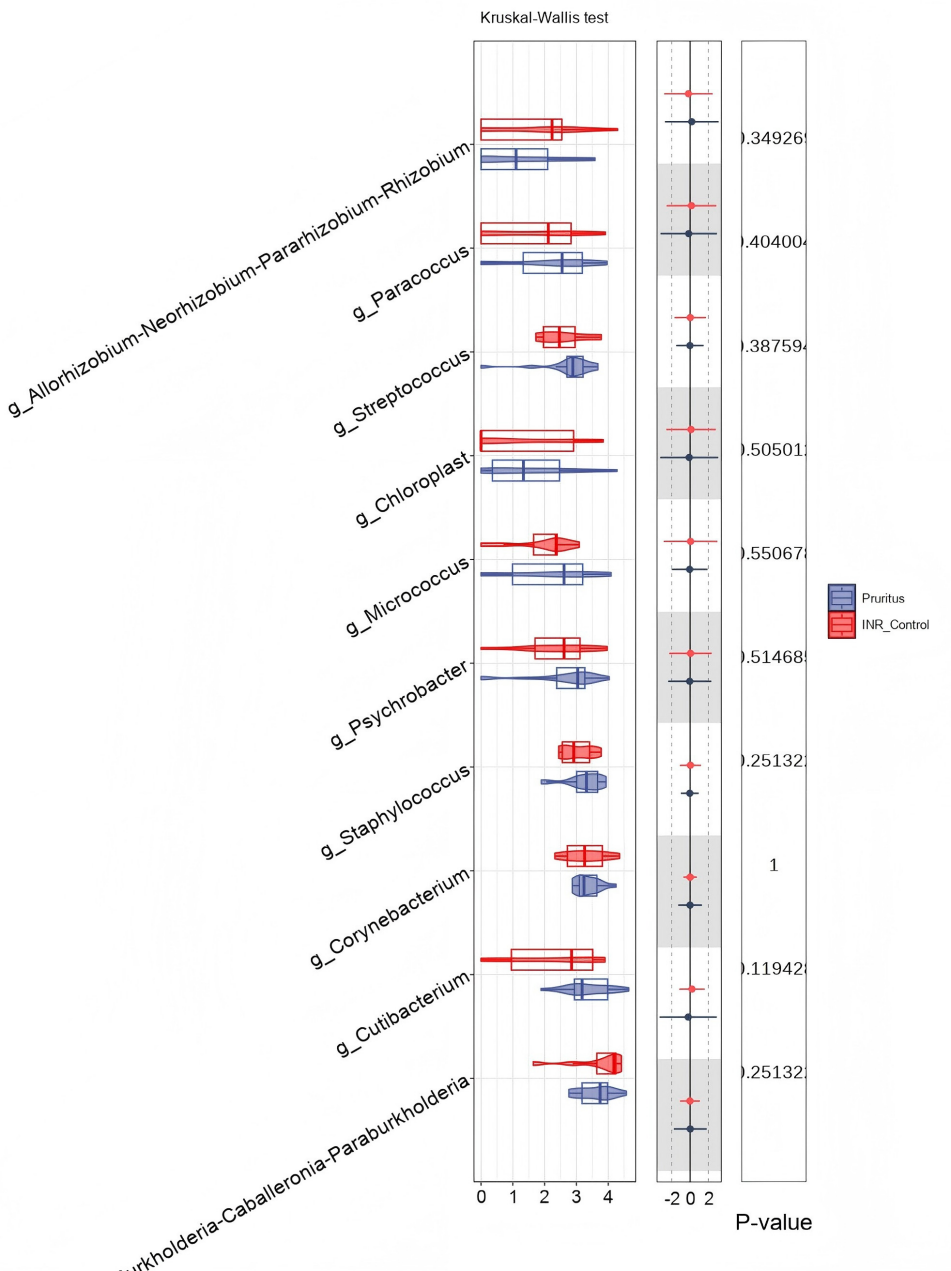
Comparison of gut microbial abundance at the Genus level. Relative abundance of listed genera is shown, and *P*-values are calculated using the Kruskal–Wallis test.

To determine species differences in the gut between the two groups, we performed a genus-level Kruskal–Wallis test. Among the listed genera, none statistically differed in relative abundance between the Pruritus and INR Control Groups (*P* > 0.05; Figure 9). In particular, the abundances of common skin commensals such as *Staphylococcus, Corynebacterium*, and *Cutibacterium* did not differ between the two groups.

**Figure 10:**
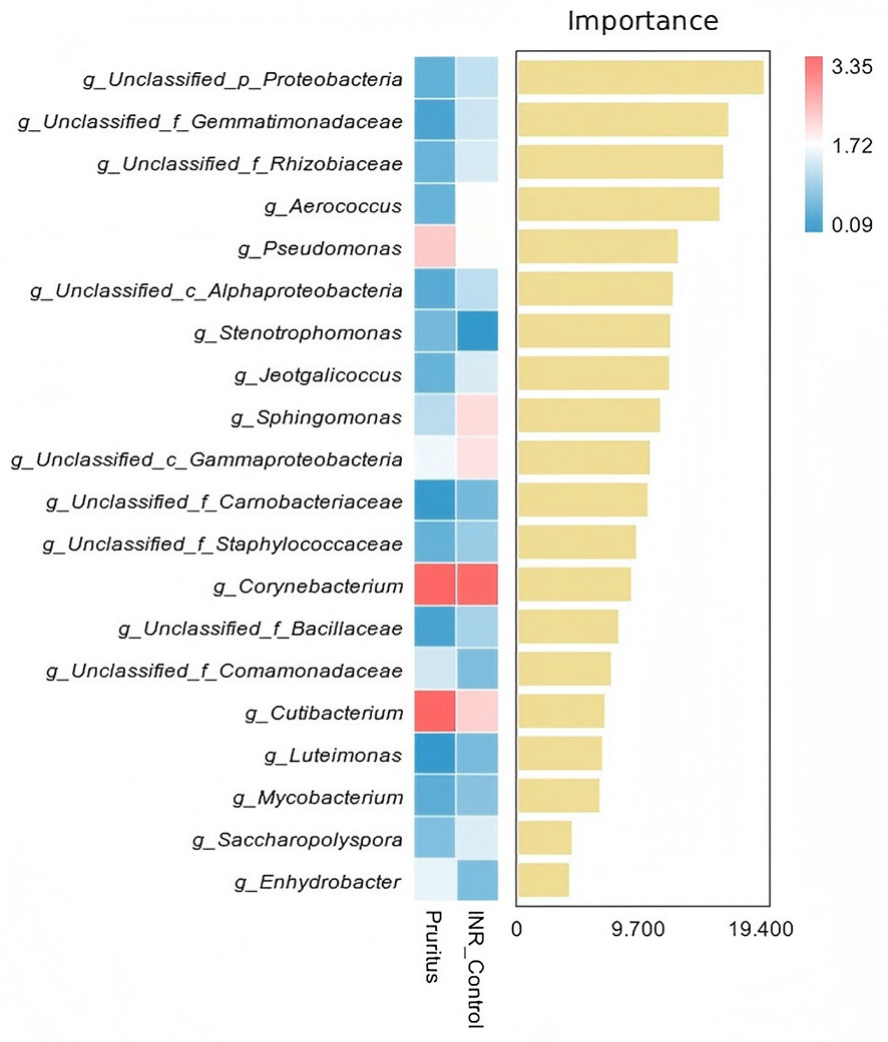
Random Forest Analysis (RFA) identifying discriminative gut microbial genera. Ranking: bacterial genera are ranked based on their Importance Score (Mean Decrease Gini) to identify potential microbial biomarkers associated with the pruritic state in PLWH with INR. Heatmap: the matrix indicates relative enrichment (Red: High; Blue: Low) in the Pruritus (*n* = 18) vs. INR Control (*n* = 15) groups.

The RFA indicated that the top five gut microbial genera ranked by importance scores were *g_Unclassified_p_Proteobacteria, g_Unclassified_f_Gemmatimonadaceae, g_Unclassified_f_ Rhizobiaceae, g_Aerococcus, and g_Pseudomonas* (Figure 10). Regarding abundance trends, the top four genera exhibited similarly low relative abundance in both groups, with no distinct differences observed visually. Among the high-ranking genera, *g_Stenotrophomonas* showed a slight enrichment in the INR Control Group compared to the Pruritus Group. Notably, the genera *g_Cutibacterium* and *g_Pseudomonas* demonstrated significant enrichment in the Pruritus Group, with *g_Cutibacterium* appearing as the most visually distinct biomarker in the heatmap. Conversely, *g_Sphingomonas, g_Unclassified_c_Gammaproteobacteria, and g_Rothia* were enriched in the INR Control Group, indicating they were ranked lower in abundance within the Pruritus Group relative to the control.

### 3.4 Relationships between the intestinal microbiota and cytokine expression

**Figure 11:**
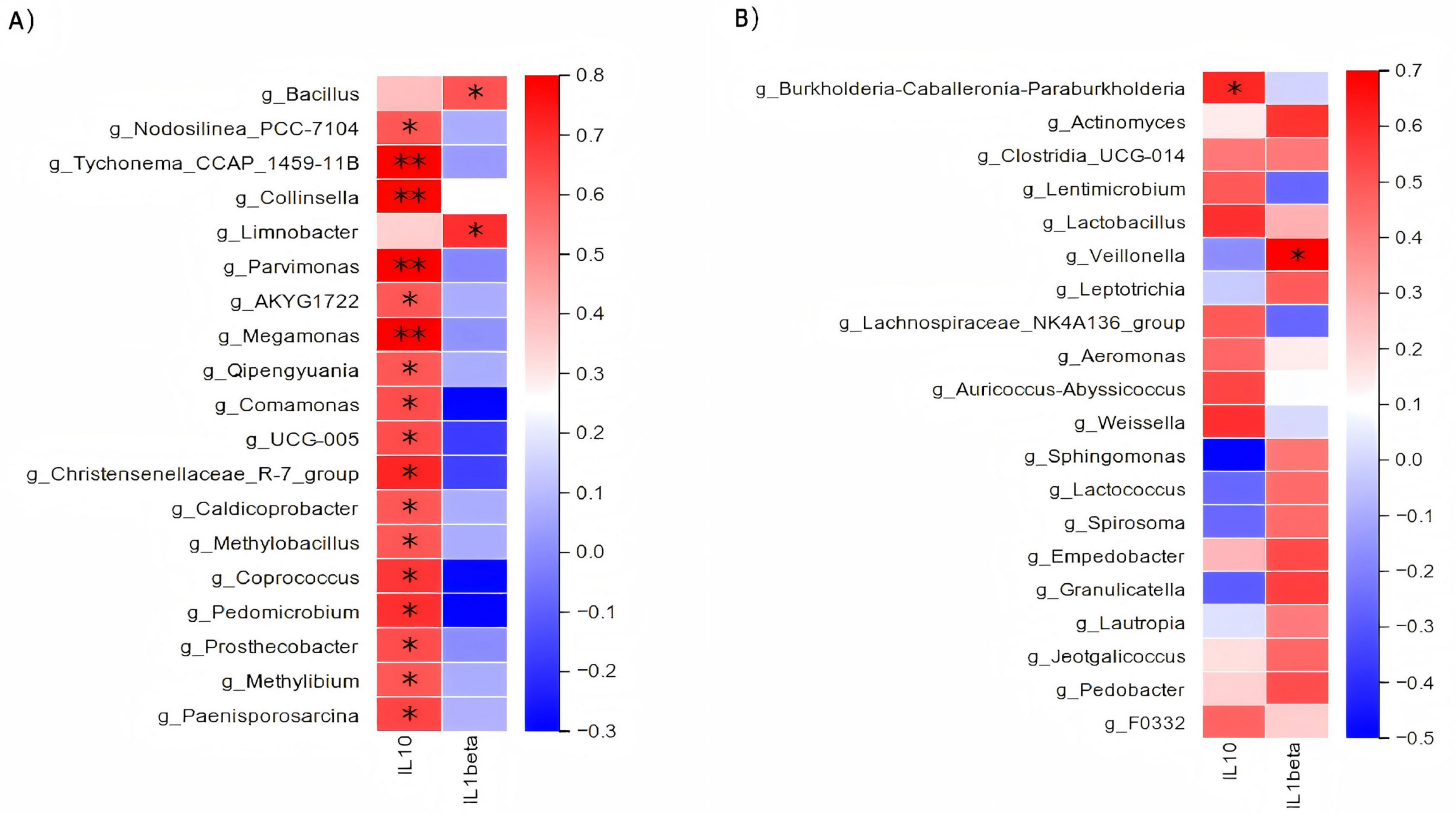
Heatmaps showing Spearman's rank correlations between microbial genera and plasma cytokines. **(A)** Correlation between skin microbiota and cytokines (IL-10 and IL-1beta). **(B)** Correlation between gut microbiota and cytokines. Correlation Scale: the color scale represents Spearman's correlation coefficient (ρ), where red indicates a positive correlation and blue indicates a negative correlation. Significance: asterisks denote statistical significance after Benjamini–Hochberg False Discovery Rate (FDR) correction: *P* < 0.05 and *P* < 0.01.

In the context of the skin microbial community, as many as 17 genera showed a significant correlation with IL-10 expression, and two genera showed a significant positive correlation with IL-1β expression (Figure 11A). Specifically, IL-1β expression showed a significant positive correlation with *g_Bacillus* (Spearman's ρ = 0.566, 95% CI: 0.135–0.817, adjusted *P* = 0.044) and *g_Limnobacter* (*adjusted P*<*0.05*). No genera were found to have a significant negative correlation with IL-1β. The anti-inflammatory cytokine IL-10 showed a significant positive correlation with 15 genera (including *g_Collinsella, g_Megamonas, and g_Parvimonas*; *P* < 0.01). IL-10 also showed a significant negative correlation with four genera (including *g_Comamonas* and *g_Coprococcus*).

Analysis of the gut microbiome (Figure 11B) revealed fewer significant correlations. IL-10 expression showed a significant positive correlation with the genus *g_Burkholderia-Caballeronia-Paraburkholderia* (*adjusted P*<*0.05*). Conversely, IL-1β exhibited a significant positive correlation with the genus *g_Veillonella* (Spearman's ρ = 0.670, 95% CI: 0.295–0.866, *P* = 0.017) No genera showed a significant negative correlation with either IL-10 or IL-1β.

## 4 Discussion

We investigated the differences in gut and skin microbial community structures and systemic inflammatory markers between PLWH who exhibit INR with and without pruritus. Our goal was to elucidate the potential microbial and inflammatory factors associated with chronic pruritus in this vulnerable population.

Our findings revealed that the skin microbiome showed significantly higher species richness via the Chao1, *Observed_species*, and *Faith_pd* indices in patients in the Pruritus vs. INR Control Group (*P* < 0.01), although community evenness, measured with the Shannon and Simpson indices, did not statistically differ. It is crucial to emphasize that this significantly higher species richness in the Pruritus Group does not equate to a healthier skin state. Instead, it likely indicates ecosystem instability and opportunistic colonization. The disruption of the skin barrier by chronic scratching may create ecological niches for transient microorganisms, leading to a “pseudo-richness” that characterizes a dysbiotic state rather than a robust microbial community. Previous studies have shown that the skin microbiome of PLWH is altered, potentially leading to skin inflammation and opportunistic infections (Ogai et al., 2023). Our findings further link this microbial dysbiosis to the clinical symptom of pruritus.

The genus *g_Bacillus* was significantly less abundant in the Pruritus Group than the INR Control Group (*adjusted P*<*0.05*). Some species within the genus *Bacillus* are considered skin probiotics, known for their role in inhibiting pathogen growth and maintaining skin barrier function (Fijan, 2014). The lower abundance of *Bacillus* in the Pruritus Group may be associated with compromised skin protective mechanisms, thereby exacerbating the onset of inflammation and pruritus. Conversely, the relative abundances of *g_Cutibacterium* and *g_Staphylococcus* were higher in the Pruritus Group than the INR Control Group, although the differences did not reach statistical significance (*P* ≥ 0.05). While *Cutibacterium* and *Staphylococcus* are common skin commensals, the abnormal enrichment of *Staphylococcus*, particularly the pathogenic species *Staphylococcus aureus*, is a significant driver of pruritic skin diseases like atopic dermatitis. This relative increase, coupled with the lower abundance of *Bacillus*, suggests a shift in the skin microenvironment of the Pruritus Group toward a more pathological microbial structure. *Cutibacterium* is a key commensal, the depletion of which has been reported in association with pruritus; therefore, even slight fluctuations in its abundance or changes in the composition of its strains may play a complex role in the pathophysiology of pruritus (Tham et al., 2022).

In terms of the gut microbiome, species richness, phylogenetic diversity, and the number of species were all significantly lower in the Pruritus Group *(adjusted P*<*0.05*). This loss of gut microbial diversity is a characteristic feature of dysbiosis in PLWH who display INR with pruritus. The persistent chronic inflammation and immune dysfunction in those with INR and pruritus may exacerbate this dysbiosis. Reduced gut microbial diversity is tightly linked to impaired intestinal barrier function and the systemic translocation of microbial products, which is a key feature associated with chronic systemic inflammation in PLWH (Rondanelli et al., 2025). Beta diversity analysis indicated that the gut microbial structure of the Pruritus Group exhibited higher inter-individual heterogeneity. Although no statistically significant differences were found in the listed genera between the Pruritus and INR Control Groups (*P* ≥ 0.05), the RFA results highlighted several genera that distinguish between the two groups. Among these, *g_Cutibacterium* and *g_Pseudomonas* were significantly more abundant in the Pruritus Group. While *Cutibacterium* and *Pseudomonas* are primarily considered skin microbes, their abnormal enrichment in the gut may suggest impaired intestinal barrier function and microbial translocation in the context of INR with pruritus, which would further exacerbate systemic inflammation. Conversely, *g_Sphingomonas, g_Unclassified_c_Gammaproteobacteria*, and *g_Rothia* were significantly less abundant in the Pruritus Group. The relative scarcity of these bacteria may represent a disruption of intestinal homeostasis, and their specific immune-regulatory functions warrant further clarification.

Through correlation analysis, this study revealed complex interactions between the skin and gut microbial communities and inflammatory cytokines, providing mechanistic insight into the systemic inflammatory basis of pruritus. In skin samples, *g_Bacillus* showed a significant positive correlation with the pro-inflammatory cytokine IL-1β. Together with the abundance data, this suggests that the lower abundance of *Bacillus* in the skin of the Pruritus Group may be related to local inflammation or be linked to a less favorable environment for *Bacillus* in an inflammatory state. In the gut microbial community, *g_Veillonella* showed a significant positive correlation with the pro-inflammatory cytokine IL-1β (*adjusted P*<*0.05*). *Veillonella* is an opportunistic pathogen, the abundance of which is high in various inflammatory diseases. This positive association suggests a potential link between this genus and systemic inflammation, though this exploratory finding requires validation in larger cohorts due to the observed error margins. Conversely, the anti-inflammatory cytokine IL-10 showed a significant positive correlation with *g_Burkholderia-Caballeronia-Paraburkholderia*. Elevated IL-10 expression may represent the body's attempt at compensatory suppression of the chronic inflammation in PLWH with INR and pruritus; this specific genus may be involved in regulating the anti-inflammatory feedback loop in the gut. This finding supports the view that the gut microbiome influences systemic immune homeostasis through metabolite production and cytokine regulation.

In conclusion, this study provides the first comprehensive characterization of the gut-skin microbiome axis in PLWH with INR who develop pruritus. We propose a unified gut-skin-immune axis model to explain the pathogenesis of this condition: chronic immune dysfunction inherent to the INR state is associated with severe gut dysbiosis—characterized by reduced diversity and enrichment of opportunistic pathogens such as *Veillonella*—which positively correlates with elevated systemic IL-1beta levels. This heightened systemic inflammatory state potentially alters the skin microenvironment, leading to the depletion of protective commensals like Bacillus and the emergence of an unstable “pseudo-richness” that collectively contributes to the manifestation of chronic pruritus. While these findings offer critical preliminary insights into the mechanisms linked to HIV-associated pruritus, several limitations must be acknowledged. As a cross-sectional study with a relatively limited sample size from a single center, our results reveal associations rather than definitive causality, and their external validity requires confirmation through larger multi-center trials. Despite these constraints, this work provides a potential foundation for future research into microbiome-targeted therapeutic strategies, such as restoring gut diversity or supplementing protective skin bacteria. Future research integrating metagenomics and metabolomics is essential to further elucidate how these differential microbes and their metabolites modulate the skin-immune interface to drive pruritus.

## Data Availability

The 16S rRNA gene sequencing data presented in the study are deposited in the NCBI BioProject repository, accession number PRJNA1433876. The data can be accessed via the following URL: https://www.ncbi.nlm.nih.gov/bioproject/PRJNA1433876.
